# Supplementary material for: Impact of Changed Use of Greenspace during COVID-19 Pandemic on Depression and Anxiety
Source: Int J Environ Res Public Health. 2021 May 29;18(11):5842. doi: 10.3390/ijerph18115842 (PMC8197797; doi:10.3390/ijerph18115842)
Supplement: Supplementary file 1 [file ijerph-18-05842-s001.zip › ijerph-1239445-supplementary.pdf]

Online supplementary materials

Impact of changed use of greenspace during COVID-19 pandemic on depression and anxiety

Seulkee Heo[1], Miraj Desai[2], Sarah R. Lowe[3], Michelle L. Bell[1]

1 School of the Environment, Yale University, New Haven, CT, USA

2 Department of Psychiatry, School of Medicine, Yale University, New Haven, CT, USA

3 Department of Social and Behavioral Sciences, School of Public Health, Yale University, New Haven, CT, USA

Contents

Supplementary Table S1. Results of bivariate analysis for potential factors and changes in visits to greenspace during the pandemic.

Supplementary Table S2. Prevalence of self-reported biggest concern in life at the time of survey, based on responses to open-ended question (n=286).

Supplementary Table S3. Prevalence of probable major depression (MD) and generalized anxiety disorder (GAD) by self-reported biggest concern in life at the time of survey (n=277).

Supplementary Table S4. Odds ratio (OR) of major depression (MD) and generalized anxiety disorder (GAD) in relation to potential confounders.

Supplementary Figure S1. Percentage of major depression (MD) and generalized anxiety disorder (GAD) cases among those who self-reported biggest concerns by type of concern. Each participant's response could include multiple types of concerns.

Supplementary Figure S2. Effect modification for the impact of decreased visits to greenspace on major depression (MD) by purposes of using greenspace, safety in neighborhood greenspace, and urbanicity.

Supplementary Table S1. Results of bivariate analysis for potential factors and changes in visits to greenspace during the pandemic.

Variable	Number of participants (%)		p-value
	Decreased visits to greenspace after COVID-19 outbreak		
	No	Yes	
Age			
19-29 years	50 (43.48)	65 (56.52)	0.001
30-49	39 (25.16)	116 (74.84)	
≥ 50	24 (46.15)	28 (63.85)	
Gender			
Men	25 (32.89)	51 (67.11)	0.745
Women	87 (35.80)	156 (64.20)	
Education			
≤ Elementary school	2 (66.67)	1 (33.33)	0.568[a]
Middle – high school	29 (35.80)	52 (64.20)	
≥ Graduate school	78 (34.82)	146 (65.18)	
Annual Income			
6 – 24 million KRW	26 (46.43)	30 (53.57)	0.046
24 – 48 million KRW	18 (24.32)	56 (75.68)	
48 – 72 million KRW	19 (30.16)	44 (69.84)	
≥ 72 million KRW	19 (39.58)	29 (60.42)	
Marital status			
Married	40 (29.41)	96 (70.59)	0.159[a]
Single	65 (39.88)	98 (60.12)	
Widowed/divorced/separated	5 (38.46)	8 (61.54)	
Urbanicity			
Urban	58 (33.0)	118 (67.0)	0.399
Rural	49 (37.70)	81 (62.3)	
Perceived safety in neighborhood greenspace			
Not safe	5 (41.67)	7 (58.33)	0.762[a]
Moderate	20 (30.30)	46 (69.70)	
Safe	45 (35.16)	83 (64.84)	
Very safe	43 (37.07)	73 (62.93)	
Importance of using greenspace in life (0: least important, 10: most important)			
Score 1 – 3	3 (50.5)	3 (50.5)	0.800[a]
Score 4 – 5	16 (31.4)	35 (68.6)	
Score 6 – 7	39 (34.8)	73 (65.2)	
Score 8 – 10	55 (35.9)	98 (64.1)	
Using greenspace for any psychological purposes			
Yes	95 (33.57)	188 (66.43)	0.172
No	18 (46.15)	21 (53.85)	
Using greenspace for any physical activities			
Yes	73 (36.87)	125 (63.13)	0.469
No	40 (32.26)	84 (67.74)	
Using greenspace for any social interactions			
Yes	61 (29.19)	148 (70.81)	0.004
No	52 (46.02)	61 (53.98)	

Forest being generally visited greenspace in 2019			
Yes	62 (31.31)	136 (68.69)	0.094
No	51 (41.13)	73 (58.87)	
Parks being generally visited greenspace in 2019			
Yes	93 (33.94)	181 (66.06)	0.384
No	20 (41.67)	181 (58.33)	
Playground being generally visited greenspace in 2019			
Yes	40 (31.01)	89 (68.99)	0.226
No	73 (37.82)	120 (62.18)	
Beach, lake, and river being generally visited greenspace in 2019			
Yes	73 (32.16)	154 (67.84)	0.115
No	40 (42.11)	55 (57.89)	
Outdoor sports field and campus yard being generally visited greenspace in 2019			
Yes	47 (31.33)	103 (68.67)	0.229
No	66 (38.37)	106 (61.63)	

Notes. [a] Fisher test was used.

Supplementary Table S2. Prevalence of self-reported biggest concern in life at the time of survey, based on responses to open-ended question (n=286).

Category of concern	N (%)
No concern	9 (3.2)
Life- or future-related concerns	77 (26.9)
Health-related concerns (including general health and COVID-19)	45 (16.0)
Job-related or financial concerns	103 (36.0)
Family-related concerns (regarding health, future, well-being)	33 (11.5)
Concerns for environment and society	19 (6.6)
Total	286 (100)

Note: Some participants’ responses noted more than one of the listed concerns.

Supplementary Table S3. Prevalence of probable major depression (MD) and generalized anxiety disorder (GAD) by self-reported biggest concern in life at the time of survey (n=277).

Category	Major Depression		Generalized Anxiety Disorder	
	Yes	No	Yes	No
Life- or future-related concerns	11 (14.3)	66 (85.7)	12 (15.6)	65 (84.4)
Health-related concerns (including general health and COVID-19)	9 (20.0)	36 (80.0)	8 (17.8)	37 (82.2)
Job-related or financial concerns	24 (23.3)	79 (76.7)	20 (19.4)	83 (80.6)
Family-related concerns (regarding health, future, well-being)	7 (21.2)	26 (78.8)	3 (9.1)	30 (90.9)
Concerns for environment and society	4 (21.1)	15 (78.9)	2 (10.5)	17 (89.5)
Total	55 (19.9)	222 (80.1)	45 (16.2)	232 (83.8)

Supplementary Table S4. Odds ratio (OR) of major depression (MD) and generalized anxiety disorder (GAD) in relation to potential confounders.

Variable	Major Depression		Generalized Anxiety Disorder	
	OR	95% CI	OR	95% CI
Age (years)				
19-29	1.00		1.00	
30-49	0.86	(0.34, 2.21)	0.77	(0.29, 2.04)
≥ 50	0.89	(0.21, 3.80)	0.67	(0.13, 3.32)
Gender				
Men	1.00		1.00	
Women	1.99	(0.65, 6.06)	1.79	(0.58, 5.49)
Smoking				
Current smoker	1.00		1.00	
Former smoker	0.56	(0.12, 2.55)	0.24	(0.04, 1.32)†
Never smoker	0.55	(0.14, 2.14)	0.61	(0.16, 2.35)
BMI‡	1.49	(1.01, 2.19)*	1.90	(1.30, 2.77)*
Education				
≤ Elementary school	1.00		-	
Middle-high school	0.40	(0.02, 6.83)		
≥ Undergraduate school	0.22	(0.01, 3.71)		
Marital status				
Married	1.00		1.00	
Single	1.52	(0.58, 4.01)	1.82	(0.65, 5.10)
Widowed/divorced/separated	4.38	(0.92, 20.8)†	2.93	(0.56, 15.48)
Experience of depression (anxiety) in last year				
No	1.00		1.00	
Yes	7.01	(3.20, 15.37)*	5.69	(2.5, 12.83)*
Not sure	3.86	(1.06, 14.01)*	1.06	(0.20, 5.60)
Having health-related concerns				
No	1.00		1.00	
Yes	1.28	(0.46, 3.58)	2.28	(0.80, 6.52)
Having job-related or financial concerns				
No	1.00		1.00	
Yes	1.62	(0.76, 3.42)	1.85	(0.84, 4.11)

Notes. *Significant at a significance level of 0.05. † Significant at a significance level of 0.10. Education was not adjusted in the model for anxiety due to over-fitting and convergence issues. ‡ OR was calculated for an interquartile range (IQR) change in BMI (4.6).

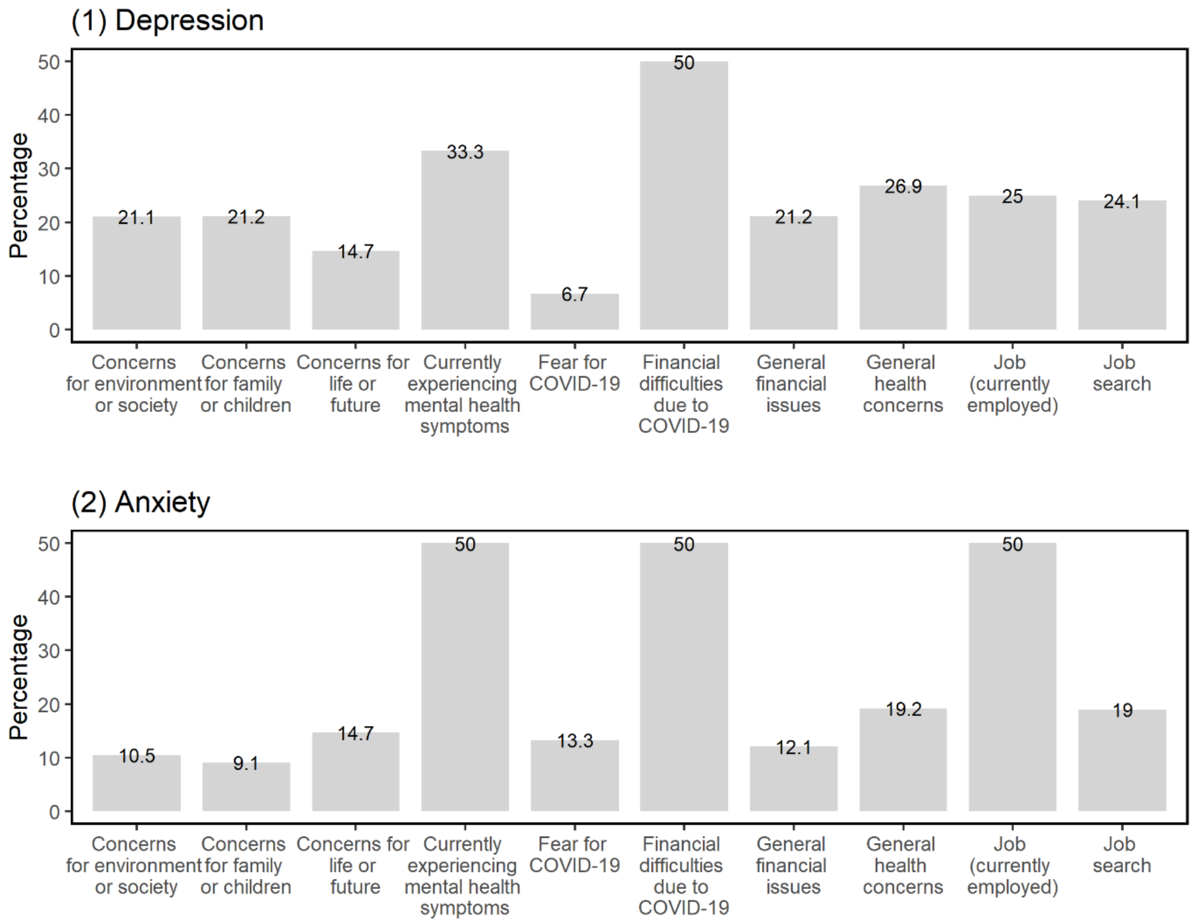

Supplementary Figure S1. Percentage of major depression (MD) and generalized anxiety disorder (GAD) cases among those who self-reported biggest concerns by type of concern. Each participant's response could include multiple types of concerns.

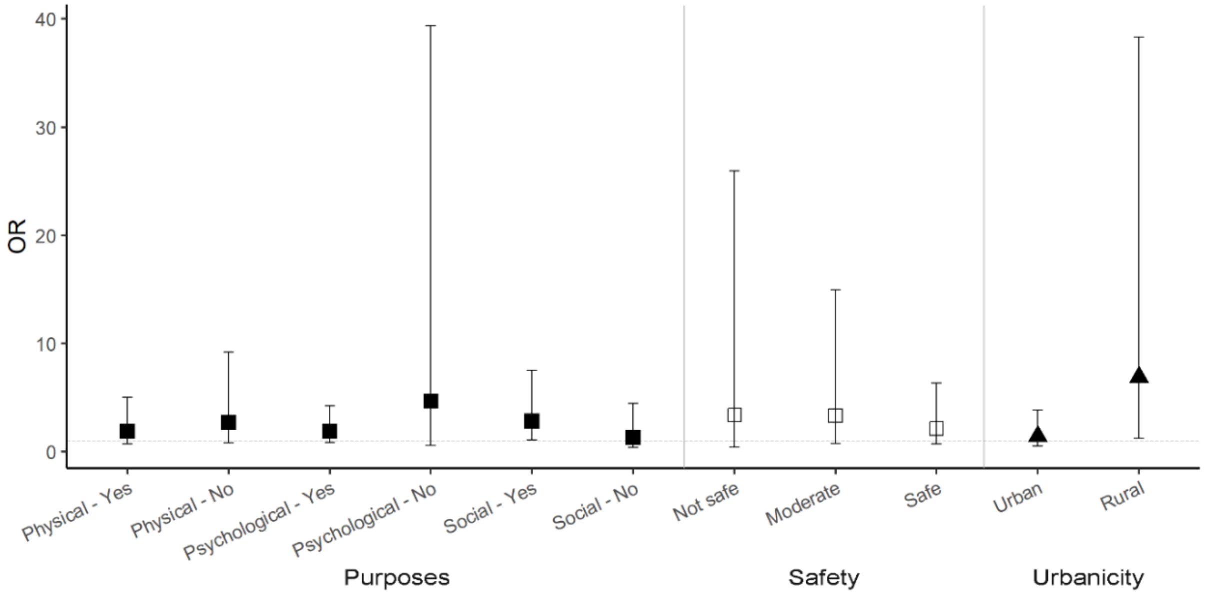

Supplementary Figure S2. Effect modification for the impact of decreased visits to greenspace on major depression (MD) by purposes of using greenspace, safety in neighborhood greenspace, and urbanicity.
